# Knee Dislocation: A Case Report, Diagnostic Vascular Work-Up, and Literature Review

**DOI:** 10.1155/2017/9745025

**Published:** 2017-02-22

**Authors:** Matthijs R. Douma, Michael D. Burg, Björn L. Dijkstra

**Affiliations:** ^1^Westfriesgasthuis, Hoorn, Netherlands; ^2^UCSF/Fresno, Fresno, CA, USA

## Abstract

Knee dislocation is an uncommon, potentially limb-threatening, knee injury. Most often caused by high-velocity trauma, it can also result from low- or even ultra-low-velocity trauma. Rapid identification of the injury, reduction, and definitive management are necessary to minimize neurovascular damage. We present a case of rotatory anterolateral knee dislocation sustained during a twisting sports-related event. Special emphasis is placed on diagnosing vascular injuries associated with knee dislocations.

## 1. Case Presentation

A 22-year-old male presented to our emergency department (ED) with acute left knee pain after a twisting injury. He sustained the injury while running during soccer, then abruptly stopping, and turning his body and upper leg with his left foot planted.

**Figure 1:**
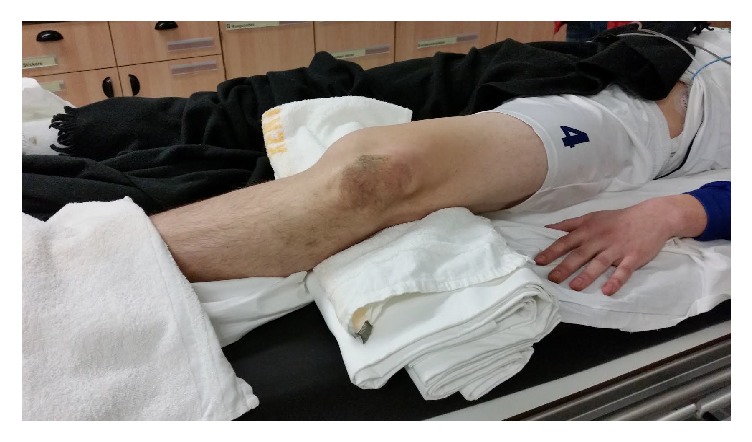
Dislocation of the left knee.

In the ED the patient offered no complaints aside from left knee pain. Vital signs were normal. Initial inspection revealed an abnormal left knee contour characterized by an anterolateral prominence below the knee (Figure 1). Physical examination showed strong palpable pedal pulses with multiphasic Doppler signals. Sensation distal to the left knee was intact. An ankle-brachial index was not done. Popliteal fossa pulse check and palpation were omitted as well. The patient's left leg was supported in a position of comfort. Range of motion testing and ligamentous stability maneuvers were not attempted due to patient discomfort.

**Figure 2:**
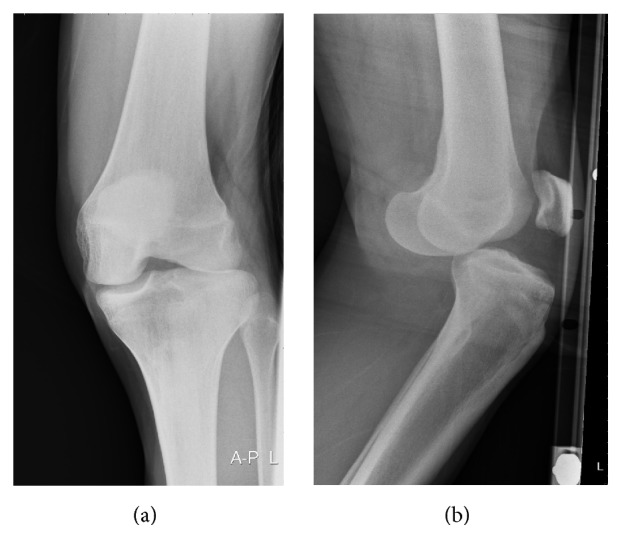
Anteroposterior and lateral radiographs showing anterolateral dislocation of the left knee.

**Figure 3:**
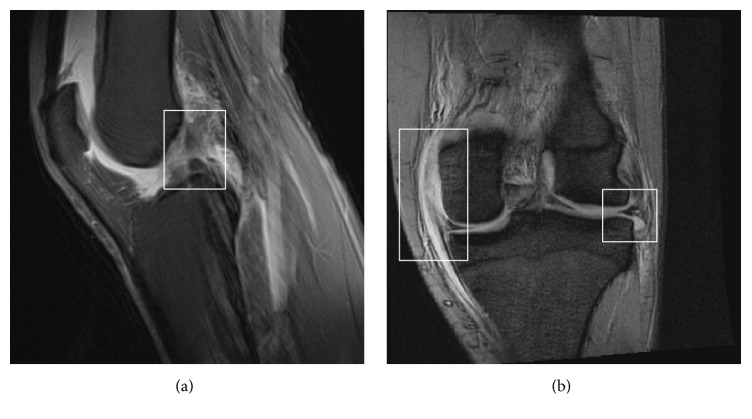
(a) MRI showing a torn anterior cruciate ligament. (b) MRI showing medial collateral ligament injury and displacement of the lateral meniscus.

Radiography confirmed the diagnosis of a rotatory anterolateral knee dislocation without fracture (Figure 2). Successful closed reduction was performed in the ED under procedural sedation. Immediate postprocedure duplex ultrasonography revealed normal patency of the popliteal fossa arteries and veins without vessel wall irregularities. No hematomas were seen. Computed tomographic angiography was not done. An in-patient magnetic resonance imaging study found a bucket handle tear of the lateral meniscus and medial collateral ligament (MCL) and anterior cruciate ligament (ACL) tears (Figure 3).

Using arthroscopy, the MCL was reattached and partial medial and lateral meniscectomies were performed. Four months following this procedure, a hamstring-transplant reconstruction of the ACL was done. The patient is recovering uneventfully.

## 2. Discussion

Compared with the entire spectrum of possible knee injuries, knee dislocation is uncommon. However, knee dislocation incidence—or perhaps just recognition—is increasing. It is widely known that a substantial percentage of dislocated knees spontaneously reduce. Additional factors contributing to the increased incidence/recognition are increased motor vehicle speeds, more high-energy sports-related falls and collisions, increased MRI use, and our population's burgeoning BMI leading to knee dislocations during routine activities of daily living. Still, an individual physician's career experience with knee dislocations may only consist of several cases. Staying current on the evolution in care of the dislocated knee is critical because of the real risk of limb loss or significant disability if management is suboptimal. In addition, the financial consequences of missing vascular injury after (spontaneously reduced) knee dislocation can be severe, as lawsuits are generally successful.

Knee dislocations can be simply defined “as ligamentous disruptions with loss of continuity of tibiofemoral articulation” [1]. However, a broader definition encompassing the entity of spontaneously reduced knee dislocations includes “those with plain radiographic tibiofemoral alignment but” with “multiple injured ligaments and gross instability on stress testing” [1]. This broader definition is important because neurovascular injury patterns are likely similar, if not identical, between acutely dislocated and spontaneously reduced but recently dislocated knees.

Knee dislocations can be classified in a variety of ways. Simply considering bony positioning yields the classification scheme of anterior, posterior, medial, lateral, combination, and rotatory with anterior being the most common form, about 40% of cases overall [2]. However, this scheme fails to account for the high percentage of spontaneously reduced knees and omits information about injury severity. Knee dislocations can also be considered as resulting from high-velocity trauma (motor vehicle crashes, falls from significant heights), low-velocity trauma (most sports injuries), and ultra-low-velocity trauma (resulting from ground-level-falls while walking or similar activities of daily living in morbidly obese individuals). Knee dislocations sustained during sports generally have a lower incidence of associated neurovascular injuries when compared with those sustained in car crashes. Oddly, the ultra-low-velocity trauma group seems to have an equal or even higher incidence of neurovascular injury when compared to high-energy dislocations [3]. The current state-of-the-art classification system is anatomic, based on the five major injury patterns that occur about the dislocated knee [1]. A full discussion of the anatomic classification is beyond the limits of this brief review but can be easily found in the medical literature [1, 4].

Of all the complications associated with knee dislocations, the direst is vascular damage, particularly popliteal artery injury. Depending on knee dislocation type, the reported incidence of any vascular injury ranges from 7 to 64% [2]. The quoted incidence of popliteal artery injury also varies widely, ranging from 32 to 45%, and spans a spectrum, from tunica intima tear to transection [5]. Additionally, the medical literature is replete with reports of amputations resulting from failure to diagnose and emergently revascularize those with knee dislocations and limb-threatening vascular injuries. One early paper describes an 86% amputation rate if devascularization persisted for more than eight hours [6]. Notably, collateral circulation around the popliteal artery is poor in most, unable to sustain distal limb viability, but robust enough to deliver palpable pedal pulses for a time, even with popliteal artery transection.

These factors led many to advocate for emergent arteriography in all patients suspected of having knee dislocations, despite the fact that many present with intact distal pulses and there are no “hard signs” of vascular damage. More recently however, a selective angiography approach has been developed and is the standard at many (but not all) medical centers.

Catheter-based angiography has been the standard test to evaluate popliteal-region vasculature. However, this test is not without risks and other downsides. False negative angiograms do occur (particularly in diagnosing intimal flaps which may thrombose during surgical repair of ligaments if a tourniquet is used). One paper cites a 4% rate of false negative angiograms [7]. False positives occur as well, ranging from a 2.4 to 7% incidence. The cost to the patient is substantial as well, cited as over $5,000 in 2003 [7]. Additionally, the patient is exposed to ionizing radiation and risks thrombosis, A-V fistula formation, bleeding, renal failure, a contrast reaction, and pseudoaneurysm formation [7]. Some of these difficulties are addressed by the use of computed tomographic angiography (CTA), which is less invasive and more time efficient and offers 100% sensitivity and specificity in detecting clinically significant arterial injury [8].

**Figure 4:**
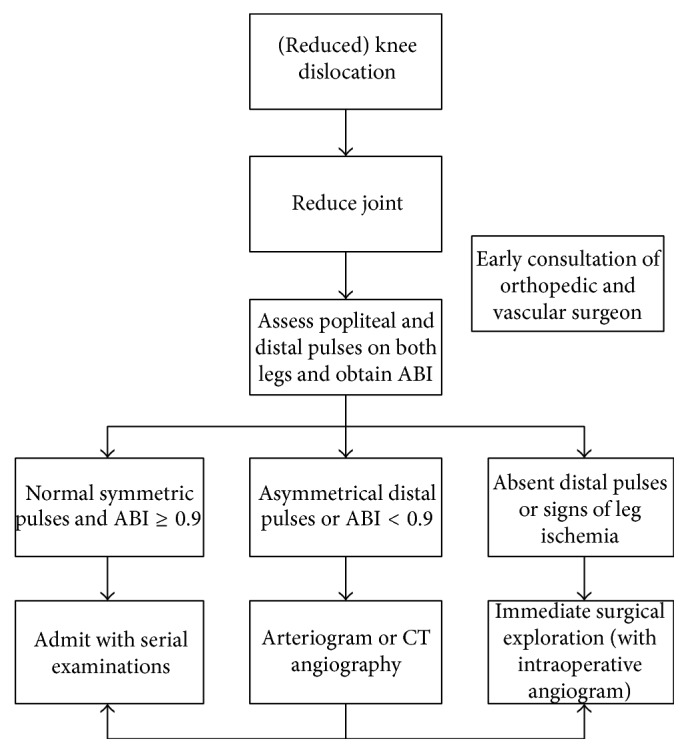
Algorithm of selective angiography approach in patients with knee dislocation (adapted from Boyce et al. [4]).

Keenly aware of the delicate risk/benefit ratio inherent in the care of the patient with a knee dislocation, many modern authors have researched various aspects of the vascular evaluation of these patients [1, 4, 9–13]. Out of these studies the so-called selective angiography approach has emerged. A 2009 protocol recommends that patients with “hard physical signs of vascular injury (hematoma, absent pulses, hemorrhage, and bruit)” undergo an immediate intraoperative angiogram [9]. Those without “vascular injury hard signs” undergo ankle-brachial index (ABI) measurement. In this group, if the ABI is <0.90, emergent angiography is done. Again, in this group, if the ABI is ≥0.90, they are admitted, studiously observed, and pulse checked frequently [9]. Other groups employ similar protocols with subtle variations [10]. All caution that conclusive evidence does not exist to support a selective angiography approach in every medical center [9, 10]. It has also been noted however that normal pedal pulses* and* an ABI ≥ 0.90 were 100% sensitive for excluding a clinically significant vascular injury [11, 13].  Figure 4 shows a flowchart of the diagnostic vascular work-up for patients with (reduced) knee dislocation.

The role of magnetic resonance angiography and duplex ultrasound for vascular evaluation has yet to be fully defined for knee dislocation patients. It seems safe to say that one must know one's medical center's imaging capabilities and expertise and apply both judgment and the available protocols when evaluating these individuals.

In retrospect, although our patient enjoyed an excellent outcome, some key elements in his care did not follow published recommendations. This occurred because both the ED attending physician and resident were seeing their first case of a knee dislocation. Thankfully, the patient was relatively low risk for a major limb-threatening vascular injury. Ideally however, once overall patient stability was assured, an immediate complete vascular examination of the patient's left leg should have been done to assess for the “hard signs” of vascular injury described above. Then an ABI should have been immediately performed. Provided that the ABI was above the threshold for immediate vascular imaging, the remainder of the work-up could have proceeded in a less urgent manner during admission, with frequent serial examinations of the affected leg.

## 3. Conclusion

Identification of knee dislocation with rapid reduction, assessment for vascular injury, and the corresponding treatment are vital to minimize neurovascular damage. We presented a summary of the current literature on the diagnostic work-up of vascular injury after knee dislocation, with emphasis on the selective angiography approach.
